# Contemporary evidence for central nervous system mechanisms underlying chronic pain perception, modulation, and chronification in adults: a scoping review

**DOI:** 10.3389/fpain.2026.1806025

**Published:** 2026-07-30

**Authors:** Annette Willgens

**Affiliations:** Nova Southeastern University - Tampa in Clearwater, Clearwater, FL, United States

**Keywords:** central nervous system, chronic pain, endocannabinnoid, mechanisms, neural circuits

## Abstract

This scoping review used the Joanna Briggs Institute guidelines and the PRISMA extension for Scoping Reviews to support transparent reporting. The eligibility criteria, search strategy, and synthesis were guided by the Population-Concept-Context framework ( 1).

## Introduction and rationale

Chronic pain neuroscience has advanced significantly in recent years, identifying central nervous system (CNS) mechanisms that contribute to pain chronification. Chronic pain is prevalent, heterogeneous, and disabling with variable trajectories and treatment responses. Current evidence highlights maladaptive reorganization across ascending and descending neural pathways, including the dorsal horn, anterior cingulate cortex, nucleus accumbens, and somatosensory cortex. Key contributors include altered neurotransmitter systems including endocannabinoid, reward and addiction circuits, and CNS structures that mediate pain perception and modulation. This review focuses exclusively on CNS mechanisms and does not address broader biopsychosocial contributors to chronic pain. The intended audience includes clinicians seeking neuroscience-based insights into adult chronic pain.

### Purpose and objectives

The purpose of this scoping review is to synthesize recent evidence on CNS mechanisms associated with chronic pain. The central question is: What CNS mechanisms underlie chronic pain perception, modulation, and chronification in adults; and how do they differ from acute and pain-free states?

### Objectives

1. Describe spinal, ascending, and dorsal horn mechanisms that contribute to chronic pain sensitization.
2. Synthesize descending, neurochemical, opioid, and endocannabinoid mechanisms involved in pain modulation.
3. Integrate supraspinal, limbic, rewards, affective, and individual-difference mechanisms into a multilevel mechanism of pain chronification.

## Methods

### Inclusion criteria

Studies were included if they:

- Focused on human adults (19 years or older)
- Studied CNS mechanisms related to chronic pain perception, modulation, or chronification
- Were published in English after 2021
- Addressed at least 1 review objective

### Exclusion criteria

Studies were excluded if they:

- Focused on pediatric populations (<19 years)
- Included acute/subacute pain, animal models, cancer pain
- Were non-English publications
- Lacked mechanistic focus or were unrelated to the review objectives.

### Information sources

Literature searches were conducted in PubMed, Embase, and Web of Science, supplemented by hand-searching reference lists. See Appendix for search strings.

### Search strategy

**Figure 1:**
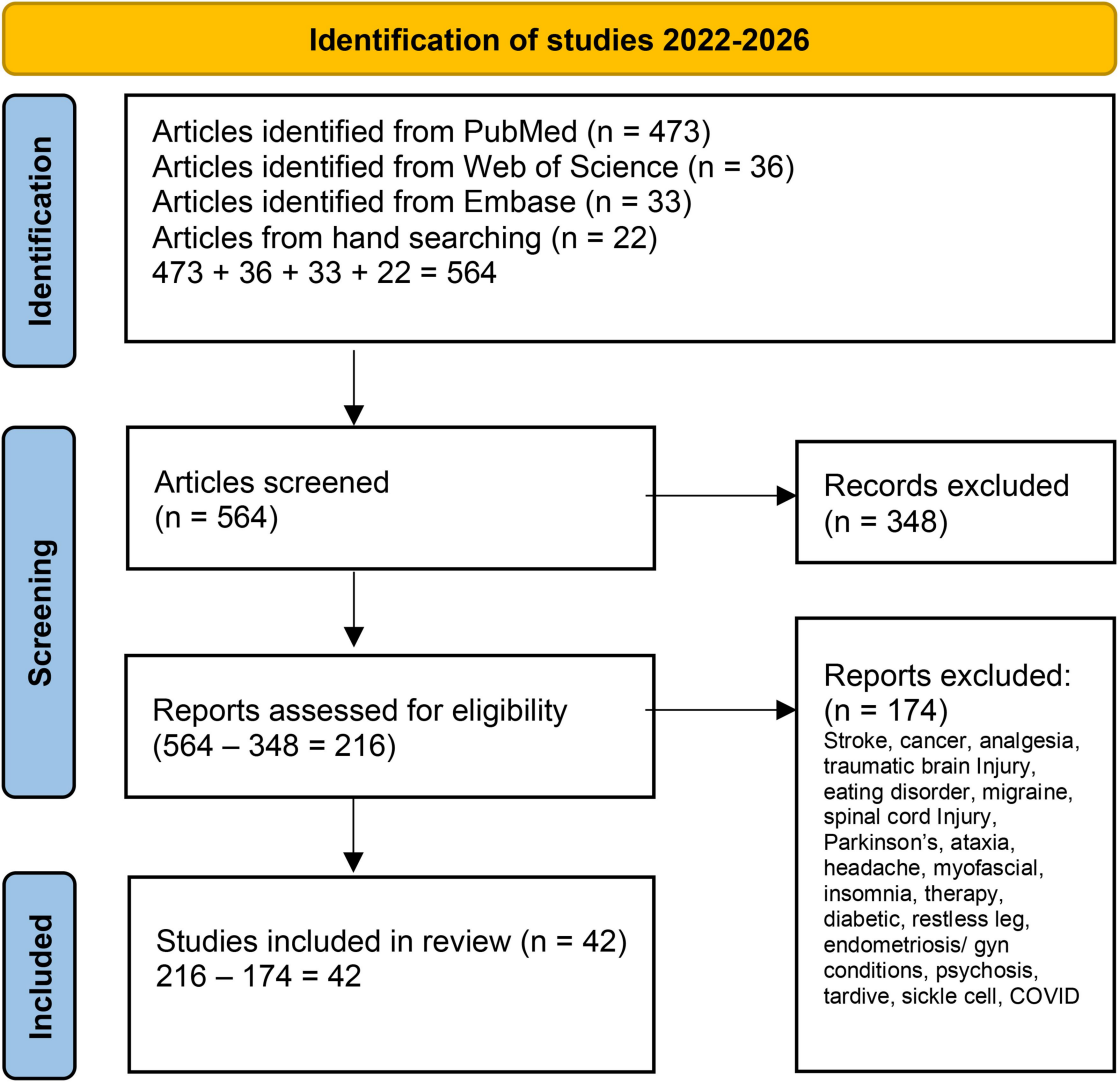
Search results.

A research librarian was consulted across several individualized meetings. Retrieved articles were screened against the inclusion and exclusion criteria and cross-checked for relevance. The screening process is summarized in Figure 1 and the Matrix Table of included studies is found in Table 1. Tables 2, 3 summarize article type and publication year distributions.

**Table 1:** Matrix table for included studies.

#	Author/Date	Purpose	Level	Grade	Method Type	Sample	Key Findings	Value in Review
1	Al-Chalabi et al. (2) 2025	To summarize the anatomy and function of the Spinothalamic Tract	5	Reference chapter	Reference chapter	N/A	This chapter provides a detailed description of the spinothalamic tract.	Foundational anatomical and functional reference for understanding spinothalamic pathways
2	Ballester et al. (3) 2022	To explore the relationship between opioid receptors and risk for opioid misuse in patients with chronic pain	2	B	PET imaging study	28 humans with chronic low back pain; 15 controls	Chronic pain participants at high risk for opioid misuse displayed higher baseline opioid receptor availability in the right amygdala; low-risk patients showed less pain-induced activation of opioid receptor transmission in the nucleus accumbens	Demonstrates biological mechanisms linking opioid receptor function to misuse risk in chronic pain patients
3	Benson et al. (4) 2024	To explain the role of dendritic spines in synaptic transmission of chronic pain	5	Narrative review	Narrative review	N/A	Dendritic spines play an important role in synaptic transmission; dendritic spines reorganize in the superficial and deeper laminae of the dorsal horn in patients with chronic pain	Explains synaptic structural changes underlying chronic pain neuroplasticity
4	Cerqueira-Nunes et al. (5) 2025	To synthesize pre-clinical evidence showing how chronic pain impairs hedonic and motivational aspects of reward.	5	Narrative review	Narrative review	N/A	Chronic pain states may trigger a persistent hypodopaminergic state across reward-system loops, contributing to anhedonia, decreased motivation, and altered reward valuation through disruptions in dopamine synthesis and opioid pathways.	Maps reward-system disruptions associated with persistent pain, particularly within the mesolimbic dopamine system.
5	Chen et al. (6) 2025	To investigate whether cortico-striatal brain circuitry changes in chronic pain extend to chronic Complex Regional Pain Syndrome (CRPS)	2	B	Resting-state fMRI connectivity study	22 patients with CRPS, controls	CRPS patients share neuroplasticity in the nucleus accumbens with other chronic pain patients, indicating a major shift in functional connectivity affecting networks involved in nociceptive and self-referential processing	Shows shared neurobiological mechanisms between different chronic pain conditions
6	Dagher et al. (7) 2024	To establish the causal effect between chronic pain and misuse of opioids and cannabis	5	Narrative review	Narrative review	N/A	Chronic pain and opioid/cannabis misuse share impaired dopaminergic transmission and poor dopamine signaling in pathways shared by opioids and cannabis	Establishes shared dopaminergic pathways between pain and substance use disorders
7	Dorado et al. (8) 2024	To investigate the connection between pain regulation and resting-state functional connectivity in older adults with chronic pain	2	B	Resting-state fMRI connectivity study	30 older adults with chronic pain, 29 pain-free older adults, 30 younger adults (all male)	Older adults with chronic pain displayed abnormal hyperconnectivity between the right dorsolateral prefrontal cortex and left amygdala; this connectivity correlated significantly with lower pain inhibition. Chronic pain during aging may lead to dysfunction of pain-inhibitory processes beyond normal aging.	Demonstrates age-related alterations in pain modulation networks in chronic pain
8	Egorova-Brumley et al. (9) 2025	To investigate the neurological links between chronic pain, sleep disturbance, and brain morphology	4	Observational	Cross-sectional, observational study	34 participants with hip osteoarthritis (OA) in “moderate” pain	Left nucleus accumbens volume was lower in patients with hip OA than normative ranges, particularly among females. Lower nucleus accumbens volume correlated directly with poorer subjective sleep (PSQI).	Shows that reduced nucleus accumbens volume may be associated with poorer sleep quality and increased pain sensitivity in hip OA.
9	España et al. (10) 2024	To review the literature on the locus coeruleus - norepinephrine inhibitory system and its influence on chronic pain pathways and comorbidities	5	Narrative review	Narrative review	N/A	In chronic pain conditions, nociceptive inputs generate neuroplastic changes in the CNS that reduce the inhibitory effects of the locus coeruleus-norepinephrine system, furthering chronic pain, anxiety, depression or sleeping disturbances	Links norepinephrine system dysfunction to chronic pain and associated comorbidities
10	Fiúza-Fernandes et al. (11) 2025	To clarify how maladaptive brain plasticity occurs in chronic pain and if common alterations exist across pathologies	4	A	Meta-analysis with qualitative synthesis	Multiple studies analyzed	Chronic pain disrupts the natural functional organization of the brain, particularly impacting default mode network (medial prefrontal cortex and precuneus), insula cortex, and descending pain modulation systems	Synthesizes evidence for common neural alterations across different chronic pain conditions
11	Flores-García et al. (12) 2023	To explore the evidence for the ventral tegmental area as a convergence ‘hub’ where pain and depression meet	5	Narrative review	Narrative review	N/A	Neural circuits involving central processing of pain and mood disorders highlight the ventral-tegmental/dopaminergic pathway as a convergence ‘hub'	Identifies common neural substrates linking pain and depression
12	Garcia-Dominguez et al. (13) 2025	To examine how pro-inflammatory markers may contribute to chronic pain, fatigue, and cognitive symptoms	5	Narrative review	Narrative review	N/A	Increased levels of interleukin-6 and TNF-alpha suggest that CNS neuroinflammation may amplify pain signaling and contribute to hyperalgesia/ allodynia.	Synthesizes evidence linking neuroinflammation to increased pain sensitization.
13	Ginsberg et al. (14) 2025	To describe subcircuits of the dorsal horn to understand how allodynia is produced	3	C	Biophysically based mathematical modeling	N/A (modeling study)	Allodynia occurs due to complex mechanisms relating to varying degrees of excitation and inhibition based on the subcircuit structure of the dorsal horn	Provides mechanistic modeling of allodynia development in dorsal horn circuitry
14	Guekos et al. (15) 2024	To determine whether central sensitization is more pronounced in women than in men	2	B	Experimental study with thermal induction and EMG	66 healthy subjects (33 women, 33 men)	Effect of thermal induction protocol was higher in women than in men; nociceptive withdrawal reflex response rate increased in women after thermal induction; central sensitization is more pronounced in women with greater supraspinal influences	Demonstrates sex differences in central sensitization mechanisms relevant to chronic pain
15	Harrison et al. (16) 2022	To describe how the periaqueductal grey is associated with endogenous pain modulation	2	B	Resting-state fMRI connectivity study	35 healthy volunteers	Some individuals are efficient pain modulators with greater functional connectivity between periaqueductal gray and pain-processing regions; heightened connectivity may contribute to beneficial clinical pain management outcomes and provide brain-based biomarkers for vulnerability or resilience to pain	Identifies periaqueductal gray connectivity as a biomarker for pain modulation efficiency and clinical outcomes
16	Hoegh (17) 2023	To explain differences between homosynaptic and hetero-synaptic plasticity and discuss clinical relevance of central sensitization and nociplastic pain	5	Editorial	Editorial	N/A	Central sensitization may explain pain during and after injury but is unlikely the single cause of chronic pain; temporal summation or hyperalgesia should not be considered direct measures of treatment effect	Provides critical perspective on limitations of central sensitization as sole mechanism in chronic pain
17	Huynh et al. (18) 2022	To identify the neural correlates of conditioned pain modulation	2	B	Conditioned pain modulation testing, voxel-based morphometryand resting-state functional connectivity	40 healthy subjects	Healthy subjects showed higher volume and stronger frontal cortex of brain regions involved with descending modulation; lateral and medial pain systems related to greater pain inhibition and facilitation during conditioned pain modulation; reveals structural and functional interactions between supraspinal areas involved in conditioned pain modulation	Establishes structural and functional neural basis of pain modulation capacity
18	Jha & Dua (19) 2025	To summarize neuroanatomy and function of the Substantia Gelatinosa of Rolando	5	Reference chapter	Reference chapter	N/A	SGR acts as a key modulatory center in pain processing by integrating peripheral inputs with descending signals, consistent with gate control theory of pain	Foundational reference for understanding spinal dorsal horn pain modulation
19	Li et al. (20) 2025	To explain the relationship between emotional disorders and chronic pain with neural circuitry and neuro-immunity pathways	5	Narrative review	Narrative review	N/A	Complex interplay between altered monoaminergic, GABAergic, and glutamatergic signaling; structural and functional changes in anterior cingulate, prefrontal cortex, insula, and locus coeruleus; involvement of neuroimmune cells and inflammatory mediators showing bidirectional relationship between chronic pain and emotional disorders	Integrates neural circuit and neuroimmune mechanisms linking pain and emotional disorders
20	Liao et al. (21) 2022	To explain the role of autophagy and apoptosis in neuropathic pain formation	5	Narrative review	Narrative review	N/A	Proinflammatory cytokines promote autophagic/apoptotic activity and neuropathic pain; autophagy may reduce proinflammatory cytokine activity and influence pain. Agents that enhance autophagy while suppressing apoptosis may help treat neuropathic pain.	Identifies cellular mechanisms (autophagy/apoptosis) relevant to neuropathic pain treatment
21	Liu et al. (22) 2025	To analyze functions of macrophages in peripheral nociceptors, dorsal root ganglia the central nervous system	5	Narrative review	Review/ perspective	N/A	Peripheral macrophages elevate nociceptor sensitivity via proinflammatory cytokine release in dorsal root ganglion; macrophages amplify pain signaling with synaptic excitability; microglia enhance pain perception by facilitating central sensitization; border-associated macrophages promote neurovascular connections and immune control	Elucidates immune cell roles in pain sensitization at peripheral and central levels
22	Löffler et al. (23) 2022	To investigate mechanisms of reward learning in patients who develop chronic low back pain	2	B	fMRI study	50 patients with chronic low back pain and pain-free controls	Connectivity between nucleus accumbens and ventromedial prefrontal cortex and reward learning independently predict transition from acute to chronic back pain	Links reward circuit dysfunction to acute-to-chronic pain transition
23	Mao et al. (24) 2025	To characterize the subdivisions of the periaqueductal gray in pain modulation of chronic low back pain	2	B	Resting-state fMRI connectivity study	75 cLBP patients, 75 healthy controls	Disrupted descending pain modulation through periaqueductal gray, post-central gyrus, and parietal lobe; impaired attention modulation through ventro-lateral periaqueductal gray, dorsomedial prefrontal cortex, pre- and post-central gyrus	Identifies specific subdivisions of the periaqueductal gray and circuits disrupted in chronic low back pain
24	Marchesini et al. 2025 (25)	To explain sex-related differences in chronic pain.	5	Narrative review	Narrative review	N/A	Chronic pain is more common in women than men with hormonal, neuroimmune, CNS, and epigenetic implications.	Generally, women experience pain sensitivity due to T cells while microglia are central contributors to men’s neuropathic pain mechanisms
25	Mathis et al. (26) 2025	To explain the neural circuits and signaling pathways of opioid use disorder	5	Narrative review	Narrative review	N/A	Opioid use disorder extends beyond the ventral tegmental-nucleus accumbens pathway; a broader system that includes prefrontal, epithalamic, and amygdala structures contributes to opioid receptor activity. Future studies should focus on location-based subcellular signaling of opioid receptors.	Expands understanding of opioid use disorder neural mechanisms beyond traditional reward pathways
26	McPherson & Ingram (27) 2022	To elucidate the cellular heterogeneity of periaqueductal gray circuits in terms of opioid regulation of descending pathway	5	Narrative review	Narrative review	N/A	Ventrolateral periaqueductal gray integrates nociceptive, cognitive, and affective processing; opioid receptors expressed on GABAergic and glutamatergic presynaptic terminals; microinjection of opioids produces analgesia; endogenous opioid effects are complex and circuit-dependent	Details cellular mechanisms of opioid analgesia in periaqueductal gray descending pain pathways
27	Medina et al. (28) 2025	To explore neural correlates of chronic low back pain symptoms and psychological therapy response using fMRI	2	B	Resting-state fMRI connectivity study	40 individuals (treated with acceptance and commitment therapy (ACT) or behavioral activation treatment for depression (BATD)	Both interventions decreased depression, psychological inflexibility, and pain catastrophizing; stronger mesolimbic-posterior insula connectivity predicted better ACT response; stronger sensory-motor network-somatosensory cortex connectivity predicted better BATD response; salience network changes correlated with psychological inflexibility across both therapies.	Identifies neural biomarkers of differential cognitive-behavioral treatment responses in chronic pain
28	Meeker et al. (29) 2022	To evaluate the effect of tonic pain on functional connectivity of descending pain modulatory network	2	B	Resting-state fMRI connectivity study	50 pain-free humans (30F) induced into tonic pain	Anterior cingulate-periaqueductal gray tonic pain connectivity positively correlates with experienced pain; amygdala network correlates with subsequently experienced pain intensity	Maps functional changes in descending pain modulation networks during acute pain
29	Mokhtar et al. (30) 2025	To summarize anatomy and function of the periaqueductal gray	5	Reference chapter	Reference chapter	N/A	Periaqueductal gray associated with learning and defensive/ aversive behavior, risk assessment, threat response, emotional/behavioral/motor/sympathetic reactions that elevate awareness; contributes to panic attacks, anxiety, and depression	Foundational reference for understanding periaqueductal gray role in pain-emotion integration
30	Nagamine (31) 2024	To determine whether gut microbiota of chronic pain patients reduces central dopamine function or has estrogen-like effects	4	A	Systematic review and meta-analysis	Multiple studies analyzed	Chronic pain patients exhibit dysbiosis with reduced alpha diversity and increased Eggerthella; reduced short-chain fatty acids weaken intestinal barrier; probiotics producing short-chain fatty acids restore dopamine function and may improve chronic pain	Links gut microbiota dysbiosis to dopaminergic dysfunction in chronic pain
31	Park et al. (32) 2022	To determine similarities in nucleus accumbens-medial prefrontal cortex circuitry across pain conditions and its prediction of outcomes	2	B	Resting-state fMRI connectivity study	50 chronic low back pain patients, 37 fibromyalgia patients, 32 controls (all male)	Reduced nucleus accumbens-medial prefrontal cortex connectivity was found in fibromyalgia and correlated with trait anxiety.	Confirms altered brain cortical-striatal neural circuits in chronic pain.
32	Peng et al. (33) 2023	To discuss PAG, locus coeruleus, parabrachial complex, hypothalamus, amygdala, and RVM roles in pain modulation and neurotransmitter influences	5	Narrative review	Narrative review	N/A	RVM as final relay of descending pain modulation receives input from PAG, amygdala, parabrachial complex, locus coeruleus, and hypothalamus; influences excitability of ON, OFF, and neutral cells with descending projections controlling spinal pain transmission; GABA, 5-HT, endogenous opioids, endogenous cannabinoids and receptors mediate facilitation/inhibition	Synthesizes understanding of descending pain modulation system and neurotransmitter regulation
33	Qiu et al (34) 2024	To explore white matter microstructure changes in ascending pain conduction pathways in chronic neck and shoulder pain (CNSP) using combined brain and spinal cord diffusion tensor imaging, and to assess correlations with clinical indicators and cognitive function.	3	Exploratory & descriptive	Diffusion tensor imaging study of the spinothalamic tract (STT)	MRI scans from 31 CNSP patients and 24 healthy controls were used to extract the spinothalamic tract (STT) and analyze fractional anisotropy (FA) and mean diffusivity (MD), which reflect nerve-fiber microstructural integrity. Findings were analyzed in relation to pain, anxiety, and depression scores.	Significant abnormalities were observed in specific segments of the bilateral cervical STT (*p* < 0.05), which were correlated with variations in pain intensity, illness duration, and levels of anxiety and depression.	Explains how chronic pain may alter STT structure and function, with potential effects on cognitive-behavioral factors.
34	Robertson et al. (35) 2023	To better understand white matter (WM) loss in chronic low back pain (cLBP)	2	B	Diffusion-weighted imaging (DWI) analysis	50 cLBP patients, 40 healthy controls	cLBP patients had lower fiber density in right anterior and bilateral superior thalamic radiations, right spinothalamic tract, right middle cerebellar peduncle, corpus callosum; higher FD in genu of corpus callosum; strong negative correlations between white matter metrics and pain catastrophizing	Links white matter structural changes to pain catastrophizing in chronic pain
35	Ryu et al. (36) 2025	To elucidate the neural mechanisms involved in stochastic (spontaneous and fluctuating) pain.	4	Translational, descriptive, prospective case series and validation study	Deep brain stimulation	7 patients experiencing chronic pain	Higher pain was associated with more regular theta and alpha activity and greater reliance on the immediately preceding state, described as a rigid, locked neural circuit. The circuit arose from one of three main pain hubs (thalamus, PAG, SCC) and projected directly to the dorsolateral prefrontal cortex in an uninterrupted, dominant pain signal.	Landmark study on how the CNS represents and transmits spontaneous (stimulus-independent) pain fluctuations.
36	Saldaña et al. (37) 2025	To explain the mechanisms of the spinal endocannabinoid system in chronic pain modulation.	5	Review of cellular, molecular, and pathophysiological mechanisms of the endocannabinoid system	Review	N/A	This review supports the concept that spinal endocannabinoid systems is a critical regulator of neuropathic pain, but its effects are context dependent.	The endo cannabinoid system may suppress pain through antinociceptive and anti-inflammatory pathways, but in chronic contexts, it may lead to hypersensitivity and persistent pain.
37	Sirucek et al. (38) 2025	To investigate whether lower excitatory/inhibitory balance exists in PAG of nonspecific cLBP and whether it relates to descending pain modulation	2	B	Magnetic resonance spectroscopy (MRS) with conditioned pain modulation testing	41 cLBP patients, 29 age and sex-matched controls	cLBP patients presented a lower glutamate + glutamine (Glx)/GABA ratio driven by decreased Glx and increased GABA. In controls, lower Glx/GABA was associated with greater pressure-pain sensitivity, but this association was absent in patients. Severe clinical pain was associated with impaired descending modulation at the hand, supporting PAG dysregulation and dysfunctional descending pain inhibition.	Demonstrates neurochemical alterations in PAG excitatory/inhibitory balance in chronic pain
38	Stensson et al. (39) 2022	To investigate the long-term effect of an Interdisciplinary Multimodal Rehabilitation Program (IMRP) for patients with chronic pain, on bioactive lipid levels and plasma telomerase activity	3	Exploratory study of an IMRP for chronic pain	Self-reports of pain, psychological distress, physical activity, and blood samples were collected before the IPRP and at a six-month follow-up. AEA, 2-AG, PEA, OEA, SEA, and telomerase activity were measured.	18 patients with complex chronic pain participating in an IPRP.	Pain intensity decreased and SEA levels increased at the six-month follow-up. Changes in SEA levels correlated significantly with pain intensity. AEA levels were inversely correlated with physical activity. 2-AG and telomerase activity were significantly correlated at the six-month follow-up.	Supports the relative effectiveness of IPRP for reducing chronic pain. SEA changes correlated with pain-intensity changes, suggesting that SEA may reflect pain-reduction effects of IPRP.
39	Terayama et al. (40) 2022	To describe interactions of primary afferents, interneurons, and glial cells causing synaptic reorganization in spinal dorsal horn after peripheral nerve injury	5	Review	Review	N/A	Spinal dorsal horn is more than a relay site; primary afferents, excitatory and inhibitory interneurons, and descending pain modulatory circuits from midbrain/medulla regulate nociceptive transmission; microglia and astrocytes involved in nociceptive transmission; functions of neurons and glial cells disrupted after peripheral nerve injury	Explains spinal dorsal horn cellular mechanisms in pain signal modification
40	Terumitsu et al. (41) 2025	To investigate correlations among functional connectivity (FC) of the salience network (SN), other neural circuits, neurometabolites, anterior cingulate cortex (ACC) measures, and Central Sensitization Inventory (CSI) scores.	3	Cross sectional, matched, case control, exploratory	Correlational Neuroimaging study	42 participants, 21 with pain, 21 without, mean age 53	Central sensitization (CS) in chronic orofacial pain may involve altered connectivity between pain-salience regions, reward circuits, basal ganglia, and attention control networks. Anterior cingulate neurometabolite correlations were associated with CSI scores in this small matched sample.	Of the regions studied, the Highlights anterior cingulate neurometabolite and connectivity findings relevant to central sensitization in chronic orofacial pain. profile confirmed altered activity.
41	Zhang et al. (42) 2025	To explain mechanisms of locus coeruleus (LC) in chronic pain and its role in modulating emotional aspects	2	B	Resting-state fMRI functional connectivity with machine learning	73 cLBP patients, 52 healthy controls	Impaired locus coeruleus-cerebellar-cortical circuits mediate modulation of emotions and chronic pain; circuits can distinguish cLBP from controls using connectivity features	Identifies locus coeruleus connectivity patterns as biomarkers for chronic pain and emotion regulation
42	Zhong & Huang (43) 2025	To systematically summarize interactions between central sensitization (CS), BDNF/TrkB signaling, and Wide Dynamic Range (WDR) neurons in neuropathic pain	5	Narrative review	Narrative review	N/A	BDNF/TrkB signaling contributes to CS by increasing WDR neuron excitability. This reinforcing role may involve non-coding RNA regulation, glial pro-/anti-inflammatory activity, and G protein-coupled receptors.	Integrates molecular signaling mechanisms (BDNF/TrkB) underlying central sensitization in neuropathic pain

**Table 2:** Distribution of included article types.

Article type	Number of articles	Included authors
Narrative Reviews/Review Articles	17	Benson; Cerqueira-Nunes; Dagher; España; Flores-García; Garcia-Dominguez; Li; Liao; Liu; Mathis; McPherson & Ingram; Peng; Terayama; Terumitsu; Zhong & Huang; Saldaña; Marchesini
Reference Chapters	3	Al-Chalabi; Jha & Dua; Mokhtar
Systematic Reviews/Meta-analyses	2	Fiúza-Fernandes; Nagamine
fMRI/Functional Connectivity Studies	10	Chen; Dorado; Harrison; Huynh; Löffler; Mao; Medina; Meeker; Park; Zhang
Other Neuroimaging/Neurophysiologic Studies	5	Ballester; Egorova-Brumley; Qiu; Robertson; Sirucek
Experimental/Modeling/Intervention Studies	4	Ginsberg; Guekos; Ryu; Stensson
Editorial/Commentary	1	Hoegh
Total	42	

**Table 3:** Article year distribution.

Publication year	Number of articles	Percent of included articles	Included authors
2025	20	50%	Al-Chalabi; Cerqueira-Nunes; Chen; Egorova-Brumley; Fiúza-Fernandes; Garcia-Dominguez; Ginsberg; Jha & Dua; Li; Liu; Mao; Mathis; Medina; Mokhtar; Ryu; Sirucek; Terumitsu; Zhang; Zhong & Huang; Saldaña; Marchesini
2024	7	16.7%	Benson; Dagher; Dorado; España; Guekos; Nagamine; Qiu
2023	5	10.0%	Flores-García; Hoegh; Peng; Robertson; Nagamine
2022	10	24.0%	Ballester; Harrison; Huynh; Liao; Löffler; McPherson & Ingram; Meeker; Park; Stensson; Terayama
Total	42	100%	

## Results

Table 4 defines key CNS structures and pathways to help readers connect neuroanatomical function with chronic pain mechanisms.

**Table 4:** Central nervous system structures related to pain chronification.

Structure/pathway	Main function	Relationship to chronic pain in the review
Spinal dorsal horn	First major CNS relay/gating site for nociceptive input; integrates peripheral afferents, interneurons, glia, and descending modulation.	Becomes hyperexcitable after injury; altered excitatory-inhibitory balance, glial activation, and synaptic remodeling contribute to central sensitization, allodynia, and hyperalgesia.
Substantia Gelatinosa/lamina II	Modulatory region within the dorsal horn that integrates peripheral input with descending signals.	Functions as an early “gate” for pain; dysfunction contributes to abnormal amplification of nociceptive input.
Anterolateral system	Ascending sensory pathway carrying pain and temperature information.	Transmits nociceptive signals toward higher brain regions; altered ascending signaling contributes to chronic pain perception.
Spinothalamic tract	Major ascending tract for nociceptive and somatosensory information.	Structural abnormalities and lower fiber density were linked with pain intensity, anxiety, depression, and catastrophizing.
Thalamus/thalamic radiations	Relay station that routes sensory information to cortical regions.	Altered thalamic pathways suggest disrupted ascending pain transmission and altered sensory-affective integration.
Somatosensory cortex	Supports conscious awareness, localization, and intensity discrimination of pain.	Chronic pain may alter somatosensory connectivity; stronger sensorimotor-somatosensory connectivity predicted response to BATD in chronic low back pain with depression.
Postcentral gyrus	Primary somatosensory cortex region; processes body-based sensory input.	PAG connectivity with postcentral regions was disrupted in chronic low back pain, suggesting altered sensory processing and descending modulation.
Precentral gyrus	Motor planning/execution region.	Mentioned in relation to disrupted PAG-linked attention/modulation circuits in chronic low back pain.
Anterior cingulate cortex	Processes affective-motivational aspects of pain, attention, and salience.	Altered ACC-PAG connectivity correlated with experienced pain; ACC changes support the emotional and attentional burden of chronic pain.
Cingulate cortex/subcallosal cingulate cortex	Integrates affect, motivation, and pain-related emotional meaning.	Identified as part of maladaptive neuroplasticity and pain-hub circuitry in chronic pain, including spontaneous pain fluctuations.
Prefrontal cortex	Supports cognitive control, appraisal, decision-making, and top-down regulation.	Chronic pain may impair reappraisal, distraction, and inhibitory control, making pain harder to suppress emotionally and cognitively.
Dorsolateral prefrontal cortex	Executive control and top-down emotion/pain regulation; linked to salience network function.	Hyperconnectivity with the amygdala was associated with lower pain inhibition in older adults with chronic pain.
Ventromedial prefrontal cortex	Value computation, meaning-making, stress regulation, and reward learning.	Connectivity with the nucleus accumbens predicted transition from acute to chronic back pain, suggesting a role in pain valuation and chronification.
Medial prefrontal cortex	Self-referential processing, regulation, and default mode network involvement.	Reduced nucleus accumbens-medial prefrontal connectivity was found in fibromyalgia and correlated with trait anxiety.
Dorsomedial prefrontal cortex	Attention, appraisal, and cognitive modulation.	Disrupted ventrolateral PAG-dorsomedial prefrontal connectivity was linked to impaired attention modulation in chronic low back pain.
Amygdala	Threat detection, emotional salience, fear, and affective pain processing.	Becomes more influential when prefrontal control is reduced; altered amygdala connectivity relates to pain inhibition, pain intensity, anxiety, and opioid misuse risk.
Insula/posterior insula	Interoception, salience, bodily awareness, and sensory-affective integration.	Altered insular activity/connectivity reflects changes in pain salience; posterior insula connectivity predicted ACT response in chronic low back pain with depression.
Precuneus	Self-referential processing and default mode network activity.	Identified as part of maladaptive neuroplasticity in chronic pain, supporting the role of self-focused processing and rumination.
Default mode network	Self-referential thought, internal attention, rumination, and autobiographical processing.	Chronic pain may shift processing toward default-mode circuitry, reinforcing negativity, self-focus, and pain-related rumination.
Salience network	Detects and prioritizes biologically important stimuli.	Dysregulation may make pain more attention-grabbing and difficult to ignore; salience network changes correlated with psychological inflexibility.
Sensorimotor network	Integrates sensory input with movement planning and body representation.	Altered sensorimotor connectivity may reflect changes in movement, body perception, and pain-related avoidance; it predicted Behavior Activation Treatment for Depression (BATD) response.
Nucleus accumbens	Reward, motivation, reinforcement learning, and valuation.	Central to pain chronification because it helps assign value to pain relief, avoidance, and reward; altered nucleus accumbens connectivity/volume was linked to chronic pain, sleep problems, anxiety, and transition to chronic back pain.
Ventral tegmental area	Dopaminergic reward hub; supports motivation and reward learning.	Identified as a convergence hub for pain and depression; disrupted dopamine signaling may contribute to anhedonia, low motivation, and pain-addiction overlap.
Mesolimbic pathway	Dopamine-based reward and motivational circuit connecting VTA, nucleus accumbens, limbic, and cortical regions.	Dysregulation contributes to altered pain valuation, avoidance reinforcement, depression, and vulnerability to opioid/cannabis misuse.
Corticostriatal/striatal circuitry	Links cortical appraisal with striatal reward/action selection.	Altered corticostriatal connectivity appears across chronic pain states and may influence nociceptive, self-referential, reward, and avoidance behaviors.
Periaqueductal gray (PAG)	Major descending pain modulation hub; integrates nociceptive, cognitive, affective, defensive, and autonomic responses.	PAG connectivity and neurochemical balance are altered in chronic pain; disrupted PAG circuits impair descending inhibition and may contribute to vulnerability or resilience.
Ventrolateral PAG (vlPAG)	PAG subdivision involved in opioid and non-opioid analgesia and descending control.	Disrupted vlPAG connectivity with prefrontal and sensorimotor regions was linked to chronic low back pain and impaired attention modulation.
Rostral ventromedial medulla (RVM)	Final common relay for descending pain modulation; contains “on”, “off”, and “neutral” cells that can facilitate or inhibit spinal nociception.	Receives input from PAG, amygdala, hypothalamus, parabrachial complex, and locus coeruleus; altered RVM output can increase or reduce spinal pain transmission.
Locus coeruleus (LC)	Major norepinephrine source; supports arousal, stress responses, and descending pain inhibition.	Chronic pain may reduce LC-norepinephrine inhibitory efficacy, worsening pain and contributing to anxiety, depression, and sleep disturbance.
Parabrachial complex/nucleus	Relays nociceptive information to limbic and autonomic centers; involved in affective-motivational pain.	Provides input to descending systems and links nociception with emotional/autonomic responses.
Hypothalamus	Regulates autonomic, endocrine, stress, and homeostatic responses.	Contributes to descending pain modulation through projections to RVM and related brainstem circuits.
Cerebellum/cerebellar-cortical circuits	Motor coordination, prediction, affective-cognitive modulation, and sensorimotor integration.	LC-cerebellar-cortical circuit dysfunction distinguished chronic low back pain from controls and was linked to emotion-pain modulation.
Middle cerebellar peduncle	White matter pathway connecting cerebellum with cortical/pontine systems.	Altered white matter metrics were reported in chronic low back pain and related to pain catastrophizing.
Corpus callosum/genu	Major interhemispheric white matter tract.	Altered fiber density in chronic low back pain suggests broader white matter reorganization associated with pain catastrophizing.
Parietal lobe	Sensory integration, attention, spatial/body representation.	Disrupted PAG-parietal connectivity was described in chronic low back pain, suggesting altered attention and sensory integration.
Epithalamic structures	Broadly involved in limbic, reward, and aversive signaling.	Mentioned as part of broader opioid-use-disorder circuitry beyond the VTA-nucleus accumbens pathway, relevant to pain-addiction overlap.
Limbic system	Emotional salience, motivation, threat appraisal, and affective learning.	Chronic pain involves altered limbic-cortical connectivity; changes may explain comorbid depression, anxiety, catastrophizing, and differential response to psychological therapies.

### Narrative synthesis

#### Objective 1. Spinal and ascending mechanisms of sensitization

The substantia gelatinosa in the spinal dorsal horn is a critical gateway for nociceptive processing because it integrates peripheral input with descending modulation (32). Rather than serving as a passive relay, this region actively regulates nociceptive signaling through interactions among excitatory interneurons, inhibitory interneurons, and glial cells (22, 40). After injury, altered primary afferent drive, remodeled interneuron connectivity, glial reactivity, and less effective descending control can shift dorsal horn transmission toward sensitization (17, 19, 20, 22). Pain signals may also arise from collaterals and spared nerves, expanding potential sources of pain beyond the original injury site (19). As a result, interneurons that normally regulate signaling may become dysfunctional and contribute directly to allodynia and hyperalgesia (13, 14).

Spinal mechanisms also differ by allodynia phenotype. Static allodynia, or pain in response to pressure or sustained stimulation, appears to result primarily from interneuron disinhibition when GABA or glycine signaling is compromised. Dynamic allodynia, or pain in response to light touch or brushing, is associated with more variable excitatory and inhibitory signaling (36). Together, these findings support mechanistic heterogeneity within dorsal horn circuitry and indicate that chronic pain presentations do not share a single spinal mechanism (4, 14, 19, 40). Disrupted GABA and glycine inhibition, glial activation, and synaptic remodeling may therefore contribute to differences in patient-reported pain intensity, anxiety, and depression (11, 14, 19, 34, 35, 40, 43).

Ascending mechanisms further extend spinal sensitization into brain-based pain processing. Nociceptive signals ascend through the spinothalamic tract, to the thalamus and somatosensory cortex, where pain is localized and processed (2). Structural abnormalities in these ascending white matter pathways, including the spinothalamic tract and thalamic radiations, have been associated with pain intensity, pain duration, anxiety, depression, and catastrophizing (36, 44). These findings suggest that chronic pain involves not only amplified spinal transmission but also altered ascending sensory pathways that interact with affective and cognitive dimensions of pain.

#### Objective 2. Descending, neurochemical, and endocannabinoid modulation

Ascending nociceptive signals interact dynamically with descending modulatory pathways, particularly through the periaqueductal gray (PAG), rostral ventromedial medulla (RVM), and locus coeruleus. These systems can suppress or amplify nociceptive transmission depending on neurotransmitter balance, stress state, and circuit context (24, 29, 38, 45). The PAG is a multifunctional hub that coordinates nociceptive, emotional, cognitive, defensive, and autonomic responses to pain (18). Distinct PAG subdivisions support opioid and non-opioid pathways, while the RVM serves as a final common relay through ON, OFF, and neutral cells that influence spinal nociception (33). Locus coeruleus-norepinephrine pathways contribute to descending inhibition, but chronic pain may reduce the inhibitory efficacy of this system and contribute to comorbid anxiety, depression, and sleep disturbance (10, 43).

Table 5 summarizes neurotransmitters and neuromodulators involved in chronic pain mechanisms, including excitatory, inhibitory, descending modulatory, reward/addiction, and endocannabinoid systems. Chronic pain is associated with increased excitatory signaling, reduced GABAergic and glycinergic inhibition, and altered descending modulation involving norepinephrine, serotonin, dopamine, endogenous opioids, and endocannabinoids. These changes contribute to central sensitization, allodynia, hyperalgesia, impaired pain inhibition, emotional pain processing, and increased vulnerability to reward/addition processes.

**Table 5:** Neurotransmitter/neuromodulator function in chronic pain mechanisms.

Neurotransmitter/neuromodulator	Area of the Brain	Function
Glutamate (20, 27)	Dorsal horn/central sensitization; PAG excitatory-inhibitory balance	Primary excitatory neurotransmitter. Increased glutamatergic signaling through NMDA/AMPA-related mechanisms contributes to dorsal horn hyperexcitability, central sensitization, allodynia, and hyperalgesia. Endocannabinoids may reduce glutamate release under typical inhibitory conditions.
GABA (33, 38)	Dorsal horn, PAG, descending modulation	Primary inhibitory neurotransmitter. Reduced GABAergic inhibition contributes to disinhibition, especially static allodynia. In the PAG, GABA can inhibit nociceptive signaling, but stress may shift this system toward hypersensitivity.
Glycine (14, 40)	Dorsal horn inhibitory circuitry	Inhibitory neurotransmitter in spinal pain modulation. Compromised glycine signaling, often paired with reduced GABA signaling, contributes to interneuron disinhibition and allodynia.
Norepinephrine (10, 33, 42)	Locus coeruleus and descending pain modulation	Strong pain-inhibitory neurotransmitter from the locus coeruleus. In chronic pain, reduced norepinephrine inhibitory efficacy may weaken descending pain control and affect limbic-emotional pain processing.
Serotonin (31)	Descending pain modulation	Modulates descending pain control. Serotonin is part of the descending system and can suppress or amplify nociceptive transmission depending on circuit context.
Dopamine (5, 7, 12, 31, 42)	Mesolimbic reward system, PAG, pain-addiction interface	Regulates reward, motivation, pain valuation, and reinforcement. Chronic pain is associated with hypodopaminergic signaling, which may contribute to anhedonia, decreased motivation, altered reward learning, opioid craving, depression, and withdrawal-like dysphoria. Dopamine may also inhibit nociceptive signaling in the PAG, though stress may reverse this effect.
Endocannabinoids — especially anandamide/AEA and 2-AG (37)	Endocannabinoid system; spinal dorsal horn; reward/addiction interface	Lipid neuromodulators involved in pain modulation. Under typical conditions, anandamide and 2-AG act through CB1/CB2 receptors to reduce glutamate and substance *P* release, dampen postsynaptic excitability, and limit glial-driven inflammation. In chronic neuropathic contexts, dysregulated endocannabinoid signaling may contribute to hypersensitivity and persistent pain.
Endogenous opioids/opioid peptides (3, 16, 27, 33)	Descending modulation; pain-addiction interface	Support descending analgesia and pain-relief valuation. Altered endogenous opioid receptor responsivity may make opioid-induced analgesia more salient and reinforcing, increasing vulnerability to craving or misuse in chronic pain.
Substance P (40, 46)	Dorsal horn/endocannabinoid discussion	Excitatory neuropeptide involved in nociceptive transmission. Endocannabinoids can reduce substance *P* release, which would dampen dorsal horn excitability and pain transmission.

The PAG is a multifunctional hub that coordinates nociceptive, emotional, and cognitive responses to pain (18). Distinct PAG subdivisions support opioid and non-opioid pathways, and stress may shift GABAergic and dopaminergic signaling from inhibitory control toward hypersensitivity (36, 38). These changes are reflected in conditioned pain modulation (CPM), a biomarker of descending inhibitory efficiency (16, 18). Since the PAG is densely connected to limbic structures involved in defensive and aversive responding, cognitive-emotional regulation may influence CPM efficiency (16). Repeated pain experience may therefore reorganize the same circuits responsible for pain modulation, helping explain why chronic pain and depression frequently co-occur and mutually exacerbate one another (27).

The endocannabinoid system may also explain how persistent pain disrupts the normal balance between excitation and inhibition. Under typical conditions, endocannabinoids act through cannabinoid receptors to reduce glutamate and substance *P* release, dampen postsynaptic excitability, and limit glial-driven inflammation. In neuropathic pain, however, this system may become dysregulated (33, 37). Dorsal horn neuron excitability is amplified by a cascade of excitatory, inhibitory, and glial mechanisms. These processes reduce GABAergic and glycinergic inhibition, and activation of microglia and astrocytes amplify dorsal horn neuron excitability (6, 20, 22). This promotes central sensitization by lowering pain thresholds and allowing normally non-painful input to produce allodynia or hyperalgesia.

**Figure 2:**
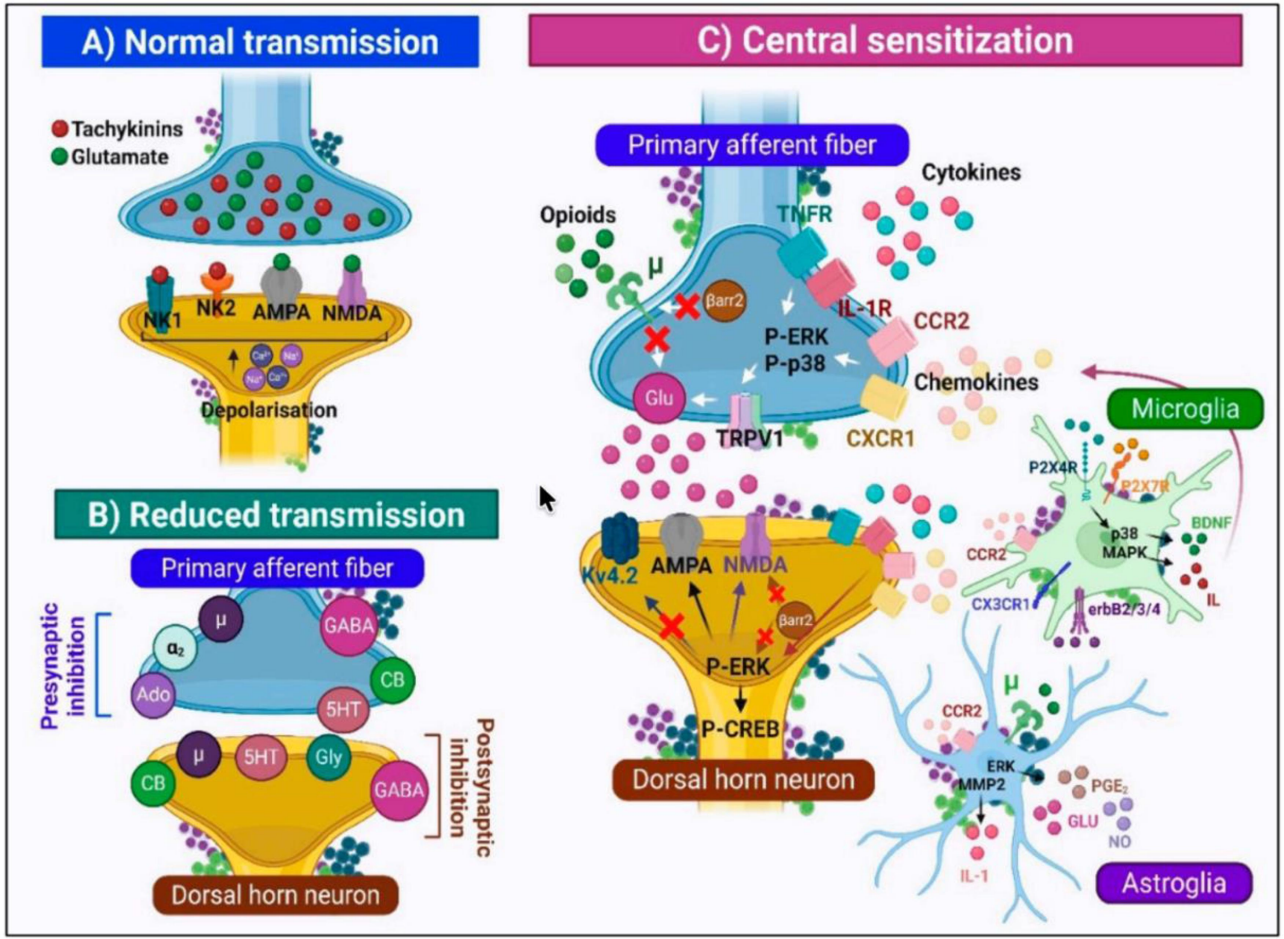
Mechanisms of nociceptive signal transmission and modulation (37). (A). Normal Transmission: Nociceptive impulses trigger release of glutamate from primary afferent terminals. Glutamate activates AMPA and NMDA receptors. Tachykinins activate G-protein signaling. (B). Reduced Transmission: Inhibitory modulation operates at presynaptic and postsynaptic levels. Presynaptically, there is reduces glutamate and substance P release. Postsynaptically, GABA_A and glycine receptors open Cl^−^ channels. (C). Central Sensitization: Presynaptically, afferents upregulate TRPV1 channels and activate cytokine/chemokine receptors, enhancing glutamate release. Neurons release microglial receptors. Postsynaptically, NMDA Mg^2+^ blockade is relieved, AMPA receptors undergo post-translational modifications to drive pro-nociceptive gene transcription. Microglia and astrocytes release pro-inflammatory mediators.

Figure 2 (37) depicts normal nociceptive transmission compared with central sensitization. In normal transmission, nociceptive signaling is mediated by specific excitatory neurotransmitters and regulated by inhibitory systems, including endocannabinoid modulation and descending pain pathways that can either inhibit or facilitate pain. In contrast, central sensitization is characterized by membrane hyperexcitability, increased neurotransmitter release, synaptic plasticity, and enhanced neuronal output. As a result, non-nociceptive afferents can activate nociceptive pathways, contributing to allodynia. Microglia are thought to initiate the sensitization process, while astrocytes may help perpetuate it.

Translational evidence also suggests that clinical improvement in chronic pain may be accompanied by measurable changes in endocannabinoid-related lipid signaling. In a small exploratory study of interdisciplinary rehabilitation, pain reduction at six-month follow-up was associated with increased lipid-endocannabinoid signaling (39). Although these findings do not directly establish CNS mechanisms, they support the possibility that rehabilitation may influence peripheral lipid biomarkers linked to pain modulation.

#### Objective 3. Supraspinal, reward, affective, and heterogeneity mechanisms

Supraspinal structures, including the amygdala, anterior cingulate cortex, prefrontal cortex, and anterior insula, integrate the sensory, emotional, and cognitive dimensions of pain (8, 11, 15, 18, 28). Chronic pain disrupts these systems through maladaptive neuroplasticity, shifting processing from salience networks toward default mode networks and reinforcing negative, self-focused reappraisal, rumination, and emotional distress (28, 41). As prefrontal inhibitory control diminishes, the amygdala may exert greater influence over threat detection and emotional reactivity to pain. This shift can impair reappraisal and descending modulation (3, 8, 29).

Reward circuits also change in relation to how individuals attend to, value, and prioritize pain (5). Altered nucleus accumbens signaling in chronic pain states may depend less on pain intensity itself than on which responses are reinforced (3, 6, 9, 32). In low back pain, connectivity between the nucleus accumbens and ventromedial prefrontal cortex, together with reward learning within these circuits, predicted the transition from acute to chronic pain (23). When reward learning is dysregulated, avoidance may become more reinforcing than engagement in valued activities, accelerating chronification (5, 23, 26). These findings suggest that chronic pain reflects not only persistent nociceptive drive and spinal sensitization, but also neuroplastic changes in systems responsible for assigning value, motivational salience, and reinforcement to pain-related behaviors.

The relationship between chronic pain and substance use disorders reflects substantial overlap between pain, reward, and addiction circuitry. Patients with chronic pain who are at elevated risk for opioid misuse demonstrate higher baseline opioid receptor availability in the right amygdala than patients at lower risk. This neurobiological difference supports a model in which altered endogenous opioid receptor responsivity changes the salience of pain relief, making opioid-induced pain relief more reinforcing (3, 26, 27). These changes also interact with prefrontal networks involved in inhibitory control and decision-making. When these networks are compromised, resistance to opioid craving may diminish (5, 23). In chronic pain states, opioid receptor activation may also produce dysphoria while triggering neuroinflammatory cascades. Activated microglia and astrocytes release cytokines and prostaglandins, which can increase excitatory glutamatergic signaling while weakening inhibitory GABAergic control (21, 22). Together, these processes contribute to a remodeled pain-reward network in which disrupted dopamine signaling may promote opioid craving, depression, and withdrawal-like dysphoria (7, 20, 23, 28).

Emerging evidence also suggests that chronic pain, substance-use vulnerability, and dopamine dysregulation may be influenced by the gut microbiome. Chronic pain has been associated with gut dysbiosis, including reduced microbial diversity, decreased short-chain fatty acid production, and weakening of the intestinal epithelial barrier (31). This increased permeability may allow bacterial products to enter circulation and contribute to systemic inflammation. Dysbiosis may also include increased Eggerthella, a bacterial genus reported to degrade dopamine (31). Because dopamine contributes to analgesic mechanisms in the PAG during nociceptive processing, reduced dopamine availability may reinforce the hypodopaminergic state associated with pain-addiction vulnerability (31).

The microbiome may intersect with sex-related pain mechanisms through hormonal, neuroimmune, inflammatory, and central pathways. For example, Eggerthella has been linked to estrogen production, which may help explain the higher incidence of chronic pain in perimenopausal and postmenopausal women. However, it remains unclear whether gut microbiota dysbiosis contributes to chronic pain primarily through reduced central dopamine function, estrogen-sensitive effects, systemic inflammation, or combined pathways (15, 25, 31).

Sex-related pain differences likely reflect interactions among hormonal fluctuations, neuroimmune signaling, central sensitization, aging, and genetic or epigenetic factors (15, 25). Estrogen and progesterone modulate nociceptive sensitivity across reproductive stages, whereas testosterone may have antinociceptive effects (25). Immune mechanisms also differ by sex, with microglia more strongly implicated in male neuropathic pain and T cells appearing more prominent in female pain sensitivity (20, 25).

Chronic pain heterogeneity is associated with measurable differences in CNS organization. Psychological intervention studies suggest that the strength and organization of nucleus accumbens connectivity may relate to whether pain is avoided or whether valued activities are maintained despite pain. When catastrophic thinking is present, including fear, rumination, or threat-based interpretations of pain, distinct patterns of neural circuitry may be observed on functional imaging. When depression is present, mesolimbic, salience, and sensorimotor circuitry may be especially relevant, and connectivity strength may help predict response to psychological interventions (5).

These circuit-level differences may explain why different psychological treatments influence pain through distinct neural pathways. In individuals receiving acceptance and commitment therapy (ACT), reductions in depression, psychological inflexibility, and pain catastrophizing were associated with changes in connectivity between the limbic system and posterior insula. In contrast, outcomes following behavioral activation treatment for depression (BATD) were associated with connectivity in the sensorimotor network and somatosensory cortex (5).

Cellular stress-response pathways may also contribute to pain persistence. Dysregulated autophagy-apoptosis signaling has been implicated in neuropathic pain, suggesting that impaired cellular homeostasis may contribute to long-term neural dysfunction and delayed restoration of normal pain processing (21). Together, these findings suggest that targeted interventions may recalibrate maladaptive pain processing by influencing the neural systems most relevant to each individual's pain-related affective, cognitive, behavioral, and biological profile.

## Discussion

### Integrated summary: A multilevel neurobiology of chronic pain

Chronic pain is characterized by distinct central nervous system (CNS) patterns, including dorsal horn sensitization, altered descending modulation, disrupted limbic-cortical connectivity, reward circuit dysregulation, and neuroimmune activation, and cellular responses to stress. These mechanisms differ from those observed in acute pain or pain-free states, occurring across spinal, brainstem, cortical, limbic, and cellular systems. However, these patterns are not uniform across chronic pain conditions, reflecting the heterogeneity of the disorder.

Chronification often begins with maladaptive spinal plasticity, in which dorsal horn sensitization amplifies nociceptive signaling This amplified signaling may persist when higher order modulatory systems fail to provide adequate inhibition. Concurrently reward and affect related circuits assign heightened emotional and motivational significance to pain, reinforcing its persistence. Immune activation and cellular stress responses further sustain these maladaptive states, while reward circuit dysregulation may increase vulnerability to both pain chronification and substance misuse.

The multilevel dysfunction underscores the need for mechanism-based interventions that address the specific CNS pathways involved in chronic pain. Taken together, this evidence supports an integrated model in which chronic pain reflects interactions among spinal sensitization, descending modulation, reward learning, affective processing, cellular stress responses, and behavioral reinforcement. Chronic pain is therefore not solely a consequence of persistent nociceptive input; it also involves maladaptive neuroplasticity in circuitry that assign meaning, value, and motivational salience to pain. These processes may be modifiable, in part, through psychologically informed interventions that target fear, avoidance, depression, inflexibility, and engagement in valued activities.

### Limitations

Limitations of this scoping review include the restriction to recent literature, and the exclusion of animal models, which may provide important mechanistic detail at the cellular level. Prioritizing contemporary adult human literature may have omitted highly relevant foundational or preclinical studies, resulting in a less comprehensive synthesis.

## Data Availability

The original contributions presented in the study are included in the article/Supplementary Material, further inquiries can be directed to the corresponding author/s.

## Appendix: Search Terms

**Pub Med**

((“Substantia Gelatinosa"[MeSH Terms] OR “Substantia Gelatinosa"[Title/Abstract] OR “lamina II"[Title/Abstract] OR “Spinal Cord Dorsal Horn"[MeSH Terms] OR “dorsal horn"[Title/Abstract] OR “Spinothalamic Tracts"[MeSH Terms] OR “spinothalamic"[Title/Abstract] OR “anterolateral system"[Title/Abstract] OR “Periaqueductal Gray"[Title/Abstract] OR “Periaqueductal Gray"[MeSH Terms] OR “rostral ventromedial medulla"[Title/Abstract] OR “Locus Coeruleus"[Title/Abstract] OR “Locus Coeruleus"[MeSH Terms] OR “descending inhibition"[Title/Abstract] OR “descending facilitation"[Title/Abstract] OR “Endocannabinoids"[MeSH Terms] OR “endocannabinoid*"[Title/Abstract] OR “receptors, cannabinoid"[MeSH Terms] OR “Nucleus Accumbens"[MeSH Terms] OR “Nucleus Accumbens"[Title/Abstract] OR “Ventral Tegmental Area"[MeSH Terms] OR “Ventral Tegmental Area"[Title/Abstract] OR “mesolimbic"[Title/Abstract] OR “dopamin*"[Title/Abstract] OR “reward circuitry"[Title/Abstract]) AND (“2021/01/20 00:00":"3000/01/01 05:00"[Date - Publication] AND “loattrfull text"[Filter]) AND ((“chronification"[Title/Abstract] OR “chronic pain"[Title/Abstract] OR “chronic pain"[MeSH Terms] OR “persistent pain"[Title/Abstract] OR “central sensitisation"[Title/Abstract] OR “neuropathic"[Title/Abstract] OR “nociplastic"[Title/Abstract]) AND (“2021/01/20 00:00":"3000/01/01 05:00"[Date - Publication]))) AND ((y_5[Filter]) AND (humans[Filter]) AND (english[Filter])) NOT “cancer” NOT “mice” NOT “rats” NOT “COVID” NOT “animal” NOT “alcohol” NOT “rodent” NOT “music” NOT “children” NOT “smoking” NOT “HIV” NOT “operative” NOT “dementia” NOT “pharm*”

**Web of Science**

TS=(“Substantia Gelatinosa” OR “lamina II” OR “Spinal Cord Dorsal Horn” OR “dorsal horn” OR “Spinothalamic Tracts” OR spinothalamic OR “anterolateral system” OR “Periaqueductal Gray” OR “Periaqueductal Grey” OR “rostral ventromedial medulla” OR RVM OR “Locus Coeruleus” OR “locus ceruleus” OR “descending inhibition” OR “descending facilitation” OR Endocannabinoids OR endocannabinoid OR “Nucleus Accumbens” OR “Ventral Tegmental Area” OR VTA OR mesolimbic OR dopamine OR “reward circuitry”) AND TS=(“chronic pain” OR “persistent pain” OR “central sensitization” OR nociplastic OR nociplasticity)

**Embase**

(‘substantia gelatinosa':ti,ab,kw OR ‘lamina II':ti,ab,kw OR ‘spinal cord dorsal horn':ti,ab,kw OR ‘dorsal horn':ti,ab,kw OR ‘spinothalamic tract':ti,ab,kw OR ‘spinothalamic tracts':ti,ab,kw OR spinothalamic:ti,ab,kw OR ‘anterolateral system':ti,ab,kw OR ‘periaqueductal gray':ti,ab,kw OR ‘periaqueductal grey':ti,ab,kw OR ‘rostral ventromedial medulla':ti,ab,kw OR RVM:ti,ab,kw OR ‘locus coeruleus':ti,ab,kw OR ‘locus ceruleus':ti,ab,kw OR ‘descending inhibition':ti,ab,kw OR ‘descending facilitation':ti,ab,kw OR endocannabinoids:ti,ab,kw OR endocannabinoid:ti,ab,kw OR ‘nucleus accumbens':ti,ab,kw OR ‘ventral tegmental area':ti,ab,kw OR VTA:ti,ab,kw OR mesolimbic:ti,ab,kw OR dopamine:ti,ab,kw OR ‘reward circuitry':ti,ab,kw)

AND

(‘chronic pain'/exp OR ‘central sensitization'/exp OR ‘chronic pain':ti,ab,kw OR ‘persistent pain':ti,ab,kw OR ‘central sensitization':ti,ab,kw OR nociplastic:ti,ab,kw OR nociplasticity:ti,ab,kw)
